# To shock or not to shock?

**DOI:** 10.1007/s12471-025-01982-z

**Published:** 2025-09-02

**Authors:** Mónica Dias, Bárbara Antunes Rocha, Sérgia Rocha, Rui Files Flores

**Affiliations:** Department of Cardiology, Hospital of Braga, Braga, Portugal

## Answer

**Fig. 1:**
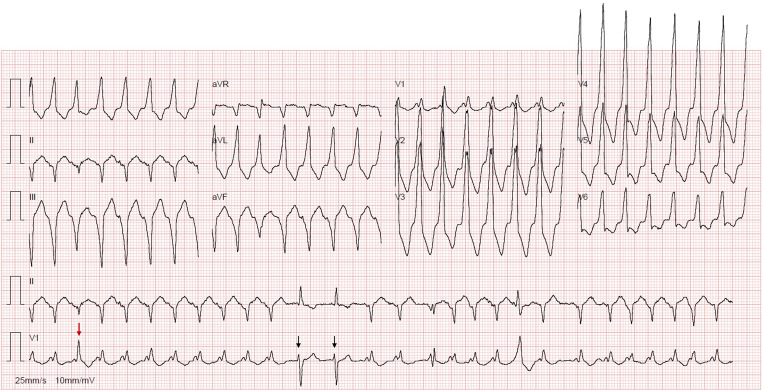
Atrial fibrillation with both pre-excited and non-pre-excited QRS complexes. Red arrow—fusion complex, combining AV nodal and accessory pathway conduction; black arrows—atrial fibrillation beats conducted via the AV node, without preexcitation

This patient presented with atrial fibrillation (AF) at a ventricular rate of ~ 190 bpm, showing both pre-excited and non-pre-excited QRS complexes. The initial ECG (Fig. 1) revealed a fast, broad, irregular rhythm consistent with AF conducting variably over an accessory pathway (AP). Notably, the degree of preexcitation remained relatively constant despite varying RR intervals, suggesting near-maximal, stable AP conduction.

Of note, the third beat (Fig. 1, red arrow) is a fusion complex, combining AV nodal and AP conduction, likely reflecting maximal preexcitation of the preceding beat. Additionally, two narrow QRS complexes (Fig. 1, black arrows) likely represent AF beats conducted exclusively via the AV node, without preexcitation.

**Fig. 2:**
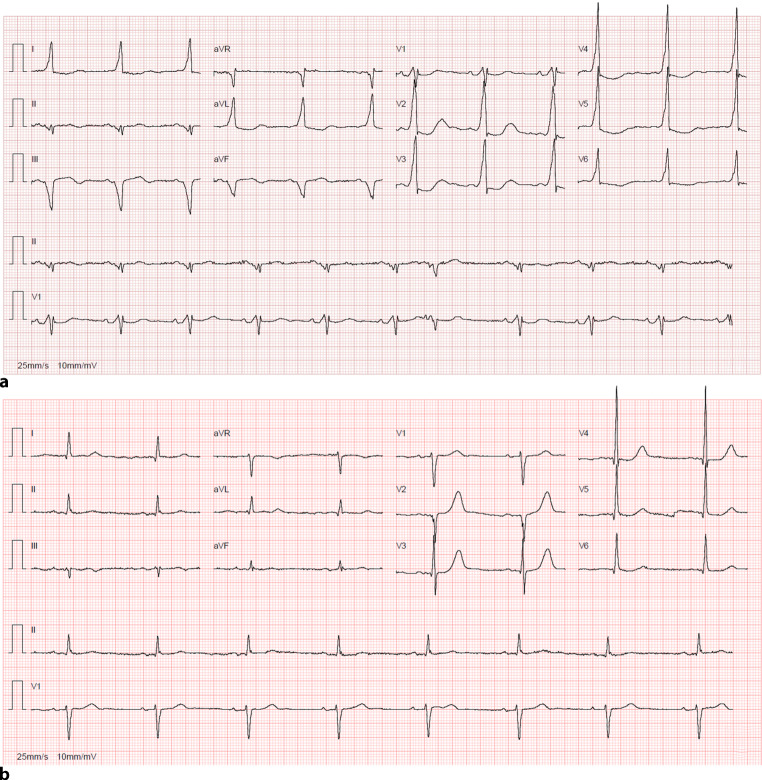
**a** Sinus rhythm after boluses of flecainide and metoprolol, with pre-excitation pattern; **b** Sinus rhythm with normal atrioventricular conduction after catheter ablation

Flecainide was administered, successfully blocking AP conduction, followed by metoprolol to reduce the ventricular rate. The patient later reverted to sinus rhythm without cardioversion (Fig. 2a). Echocardiography showed moderate LV systolic dysfunction, which recovered within four days. Electrophysiology study confirmed a right-sided AP, and catheter ablation was successfully performed (Fig. 2b).

Pre-excited AF with rapid AP conduction poses a high risk of ventricular fibrillation. In stable patients, AP-blocking agents like flecainide or procainamide are preferred over AV nodal blockers, which may paradoxically enhance AP conduction [1]. However, when preexcitation is already maximal—as in this patient—the incremental risk posed by AV nodal blockade may be less significant.

Rapid ventricular rates and electrical desynchrony may result in transient LV dysfunction, which warrants reassessment following rhythm stabilization [2]. New-onset pre-excitation in elderly patients should not be overlooked, particularly when presenting with arrhythmias and heart failure [3].
